# Association between postoperative complications and delayed adjuvant therapy following total laryngectomy: a retrospective cohort study

**DOI:** 10.25122/jml-2026-0089

**Published:** 2026-09

**Authors:** Bianca Petra Taher, Serban Vifor Gabriel Bertesteanu, Raluca Grigore, Alexandru Nicolaescu, Gloria Simona Bertesteanu, Teodora Elena Schipor-Diaconu, Mihai Dumitru Tudosie, Irina Doinita Popescu, Bogdan Popescu, Paula Luiza Bejenaru, Alexandra Marin, Anca Ionela Cîrstea, Simona-Andreea Rujan

**Affiliations:** 1 Department 12-Otorhinolaryngology, Ophthalmology, Faculty of Medicine, Carol Davila University of Medicine and Pharmacy, Bucharest, Romania; 2 Department of ENT, Head and Neck Surgery, Colțea Clinical Hospital, Bucharest, Romania; 3 Prof. Dr. Dimitrie Gerota Emergency Hospital, Bucharest, Romania; 4 Central Military Emergency Hospital Carol Davila, Bucharest, Romania

**Keywords:** laryngeal neoplasms, laryngectomy, postoperative complications, radiotherapy, adjuvant, time-to-treatment, pharyngocutaneous fistula, head and neck neoplasms, AJCC, American Joint Committee on Cancer, BMI, body mass index, CI, confidence interval, CRT, chemoradiotherapy, HNSCC, head and neck squamous cell carcinoma, IQR, interquartile range, LSCC, laryngeal squamous cell carcinoma, OR, odds ratio, PCF, pharyngocutaneous fistula, POCRT, postoperative concurrent chemoradiotherapy, PORT, postoperative radiotherapy, RT, radiotherapy, SD, standard deviation, RR, relative risk, STROBE, Strengthening the Reporting of Observational Studies in Epidemiology, TL, total laryngectomy, TTI, time-to-treatment interval

## Abstract

Total laryngectomy (TL) remains an essential treatment for selected patients with advanced laryngeal squamous cell carcinoma (LSCC), but postoperative complications may delay adjuvant radiotherapy (RT) or chemoradiotherapy (CRT). This retrospective cohort study evaluated the association between postoperative complications and time to adjuvant therapy after primary TL. The analytic cohort included patients who underwent primary TL for LSCC between January 2022 and December 2025 at a tertiary university hospital, had an indication for postoperative RT or CRT, initiated adjuvant therapy, and had a calculable time-to-treatment interval (TTI). Complications occurring before adjuvant treatment initiation were recorded and graded using the Clavien-Dindo classification. Delayed adjuvant therapy was defined as RT/CRT initiation more than 42 days after surgery; delay beyond 56 days was also assessed. Among 203 patients, postoperative complications occurred in 49 (24.1%), while 154 (75.9%) had an uncomplicated course. Median TTI was longer in patients with complications than in those without complications (56 vs 42 days; *P* < 0.001). Delay beyond 42 days occurred in 47/49 patients (95.9%) with complications versus 30/154 patients (19.5%) without complications (relative risk, 4.92; 95% CI, 3.55–6.82; *P* < 0.001). Delay beyond 56 days occurred in 23/49 patients (46.9%) with complications and in no uncomplicated patient. Pharyngocutaneous fistula, wound-related complications, and major complications requiring reintervention were associated with the longest treatment delays. Postoperative complications after primary TL were strongly associated with delayed initiation of adjuvant therapy, supporting prevention, early recognition, and prompt management of complications as important components of multidisciplinary oncological care.

## INTRODUCTION

Laryngeal squamous cell carcinoma (LSCC) remains a clinically important malignancy of the upper aerodigestive tract, with a substantial burden in terms of morbidity, functional impairment, and cancer-related mortality [1]. Treatment selection is primarily driven by tumor stage, anatomical extension, laryngeal function, patient-related factors, and multidisciplinary assessment, and may include surgery, radiotherapy, concurrent chemoradiotherapy, or combined-modality treatment [2]. Although organ-preservation strategies have become increasingly integrated into the management of locally advanced laryngeal cancer, total laryngectomy (TL) remains an essential curative procedure for selected patients with advanced disease, particularly when functional laryngeal preservation is not feasible or when surgical treatment is considered the most appropriate oncological option [3].

Despite its established oncological role, TL is associated with considerable postoperative morbidity. Wound complications, pharyngocutaneous fistula, infection, dehiscence, hemorrhage, pulmonary complications, prolonged hospitalization, and readmission are among the most relevant adverse postoperative outcomes after TL [4]. Among these, pharyngocutaneous fistula (PCF) is one of the most frequent and clinically consequential complications, because it reflects a failure of pharyngeal closure and may lead to salivary leakage, local infection, prolonged enteral feeding, delayed oral intake, repeated wound care, reoperation, and impaired quality of life [5]. Several patient-, tumor-, and treatment-related factors have been associated with the development of PCF and other postoperative complications, including nutritional impairment, anemia, prior radiotherapy, comorbidities, surgical extent, and reconstructive technique [5–9].

The clinical relevance of postoperative complications extends beyond immediate surgical morbidity. Patients undergoing TL for advanced LSCC frequently present with adverse pathological features that indicate the need for postoperative radiotherapy (PORT) or postoperative concurrent chemoradiotherapy (POCRT), including advanced primary tumor stage, nodal disease, extranodal extension, positive or close surgical margins, perineural invasion, and lymphovascular invasion [10]. In this setting, adjuvant therapy is not simply an additional treatment step, but a critical component of curative-intent oncological management [10,11]. Therefore, any postoperative event that interferes with wound healing or delays referral, planning, simulation, or initiation of PORT/POCRT may have oncologically relevant consequences, not merely surgical ones.

Timely initiation of PORT is widely recognized as a key quality indicator in head and neck squamous cell carcinoma (HNSCC). A time interval of no more than 6 weeks (42 days) between surgery and the start of PORT is commonly used as the recommended postoperative treatment window and has been incorporated into quality-of-care discussions and benchmarking efforts [12,13]. This interval is clinically important because prolonged delays may allow accelerated tumor cell repopulation and may reduce the effectiveness of postoperative irradiation, particularly in patients with high-risk pathological features [14]. Recent systematic reviews and meta-analyses have shown that delayed PORT is associated with worse oncological outcomes in patients with head and neck cancer, including poorer overall survival, disease-free survival, recurrence-free survival, and locoregional control [13–15].

The causes of delayed PORT are multifactorial and include patient-related factors, comorbidity, social barriers, referral pathways, postoperative recovery, dental evaluation, radiation planning logistics, and health-system factors [14,16]. However, postoperative complications represent a particularly actionable cause of delay because they are directly linked to surgical risk stratification, perioperative optimization, wound surveillance, and early complication management [17,18]. In patients who develop PCF, dehiscence, infection, or hemorrhage, the clinical team may be forced to balance two competing priorities: ensuring adequate wound healing before irradiation and preserving the oncologically recommended adjuvant treatment window. This tension is especially relevant after TL, where pharyngeal wound breakdown and salivary contamination may make early irradiation unsafe or impractical.

Although the association between postoperative complications and delayed adjuvant therapy is clinically plausible, the available literature remains limited in several important respects. Many studies evaluating PORT delay include heterogeneous HNSCC populations, pooling oral cavity, oropharyngeal, hypopharyngeal, and laryngeal tumors, as well as different surgical procedures with substantially different complication profiles [13–16]. In addition, most studies focus on predictors of PORT delay at the health-system or population level rather than quantifying the direct effect of specific postoperative complications after TL. As a result, TL-specific evidence remains limited on how often complications push patients beyond the 42-day threshold, how long the delay is in absolute days, which complication types are most strongly associated with delayed adjuvant therapy, and whether complication severity correlates with the magnitude of delay.

This gap is clinically relevant because identifying postoperative complications as a measurable cause of delayed adjuvant therapy reframes complication prevention as an oncological priority. If specific complications are shown to prolong the time-to-treatment interval beyond the recommended window, then preoperative optimization, risk-adapted surgical planning, early recognition of wound breakdown, timely reintervention, and coordinated referral to radiation oncology may become essential components of oncological quality improvement after TL. Such evidence may also support more accurate preoperative counseling and more structured postoperative pathways for high-risk patients.

This study aimed to evaluate the impact of postoperative complications on the timing of adjuvant therapy initiation in patients undergoing primary total laryngectomy for laryngeal squamous cell carcinoma at a tertiary teaching hospital. The primary objective was to determine whether postoperative complications were associated with delayed initiation of adjuvant therapy, defined as a time-to-treatment interval greater than 42 days from surgery to the first day of PORT or POCRT. Secondary objectives were to compare the time-to-treatment interval between patients with and without complications, determine the proportion of patients exceeding both 42-day and 56-day thresholds, quantify delays by complication type, and explore the relationship between complication severity and adjuvant therapy delay.

## MATERIAL AND METHODS

### Study design, setting, and reporting framework

This was a single-center retrospective cohort study conducted in the Department of Otorhinolaryngology and Head and Neck Surgery of Colțea Clinical Hospital, Bucharest, Romania, a tertiary public university teaching hospital affiliated with Carol Davila University of Medicine and Pharmacy. The study included an analytic cohort of patients recorded in the institutional database who underwent primary total laryngectomy for laryngeal squamous cell carcinoma between January 2022 and December 2025, had an indication for postoperative radiotherapy or concurrent chemoradiotherapy, initiated adjuvant therapy, and had sufficient documentation to calculate the time-to-treatment interval. Because the available retrospective database was constructed from patients with calculable adjuvant therapy initiation dates, the present analysis should be interpreted as a treatment-initiator cohort rather than as a complete screening cohort of all total laryngectomy patients treated during the study period. The manuscript was prepared in accordance with the Strengthening the Reporting of Observational Studies in Epidemiology (STROBE) recommendations for observational cohort studies [19].

The study was designed to evaluate whether postoperative complications occurring after primary total laryngectomy and before the initiation of adjuvant therapy were associated with delayed start of postoperative radiotherapy or postoperative concurrent chemoradiotherapy. The exposure of interest was the occurrence of at least one postoperative complication, and the main outcome was delayed initiation of adjuvant therapy, defined as more than 42 days from surgery to the first day of adjuvant treatment.

### Patient selection

Patients were eligible for the analytic cohort if they met all of the following criteria: histologically confirmed primary laryngeal squamous cell carcinoma; treatment by primary total laryngectomy with curative intent; postoperative indication for adjuvant radiotherapy or concurrent chemoradiotherapy as determined by the multidisciplinary tumor board; initiation of adjuvant treatment; and complete documentation of both the date of surgery and the date of first postoperative radiotherapy fraction.

Patients were not included in the analytic cohort if they underwent salvage total laryngectomy after previous radiotherapy, chemotherapy, chemoradiotherapy, targeted therapy, or immunotherapy for head and neck cancer; had distant metastatic disease at the time of surgery; had synchronous malignancy requiring competing oncological treatment; underwent palliative surgery; had no indication for adjuvant therapy; or lacked sufficient documentation to calculate the time-to-treatment interval.

The available retrospective dataset did not contain a complete screening log for all total laryngectomy procedures performed during the study period. Therefore, the number of patients excluded at each pre-analytic eligibility step could not be reconstructed reliably. This limitation was considered when interpreting the representativeness of the analytic cohort.

### Multidisciplinary treatment pathway

All patients were evaluated and managed within a multidisciplinary framework involving head and neck surgeons, medical oncologists, radiation oncologists, radiologists, pathologists, anesthesiologists, and other specialists when clinically required. The indication for total laryngectomy was determined by tumor extent, functional status, patient-related factors, and curative treatment intent. Following surgery, final histopathological findings were reviewed, and the indication for adjuvant radiotherapy or concurrent chemoradiotherapy was determined by the multidisciplinary tumor board. In general, postoperative radiotherapy was considered for patients with adverse pathological features such as advanced pT category, nodal disease, close or positive surgical margins, perineural invasion, lymphovascular invasion, or other high-risk features identified by the tumor board. Concurrent chemoradiotherapy was generally considered for patients with very high-risk features such as positive margins and/or extranodal extension when documented. However, detailed pathological variables such as margin distance, extranodal extension, perineural invasion, and lymphovascular invasion were not consistently available in the retrospective analytic dataset and were therefore not included in the statistical models.

Adjuvant treatment was defined as postoperative radiotherapy alone or postoperative concurrent chemoradiotherapy. The start of adjuvant therapy was defined as the first day of postoperative radiotherapy, corresponding to the first delivered radiation fraction. For patients receiving concurrent chemoradiotherapy, the same definition was used, because the time-to-treatment interval was anchored to the start of postoperative irradiation.

### Data sources and collected variables

Data were extracted retrospectively from electronic medical records, paper-based hospital charts, operative reports, pathology reports, discharge summaries, and oncology/radiotherapy records. All extracted data were entered into a structured database using anonymized patient identifiers. The database was checked for duplicate entries, inconsistent categorical labels, impossible dates, and missing values before statistical analysis.

Patient-related variables included age at surgery, sex, body mass index (BMI), and smoking status. Tumor-related variables included postoperative T category, N category, and overall AJCC stage according to the eighth edition of the American Joint Committee on Cancer staging system. Treatment-related variables included date of total laryngectomy, date of adjuvant therapy initiation, time-to-treatment interval, and available information regarding adjuvant treatment.

Postoperative variables included the occurrence of any complication before the initiation of adjuvant therapy, type of complication, Clavien-Dindo severity grade, need for surgical reintervention, and return to the operating room. Complications were recorded both as individual events and as a binary patient-level variable indicating the presence or absence of at least one postoperative complication.

### Definition and classification of postoperative complications

A postoperative complication was defined as any clinically documented adverse event occurring after total laryngectomy and before the initiation of adjuvant therapy that required clinical observation, pharmacological treatment, wound care, prolonged enteral feeding, procedural intervention, readmission, or surgical reintervention. This exposure window was selected because the study focused on complications documented during the postoperative interval in which readiness for adjuvant treatment was assessed. Exact dates of onset were not consistently available for all complication types; therefore, a fixed 30-day postoperative complication ascertainment window and a 30-day sensitivity analysis could not be performed. The possibility of time-window bias was addressed in the interpretation of the findings.

The predefined complication categories included pharyngocutaneous fistula, wound infection, wound dehiscence, flap dehiscence or flap necrosis, postoperative hemorrhage, and other clinically relevant postoperative adverse events documented in the medical records. Pharyngocutaneous fistula was defined as a pathological communication between the pharyngeal lumen and the cervical skin, with salivary leakage confirmed clinically and/or radiologically when imaging was used. Wound infection was defined as a postoperative cervical wound infection requiring antibiotic treatment, local wound care, drainage, or further intervention. Dehiscence was defined as partial or complete disruption of the surgical wound or flap closure. Postoperative hemorrhage was defined as clinically significant bleeding requiring observation, transfusion, procedural management, surgical exploration, or vascular control.

Complication severity was graded according to the Clavien-Dindo classification, which categorizes postoperative complications according to the therapeutic intervention required to treat them [20]. For patient-level analyses, the highest Clavien-Dindo grade recorded for each patient was used. Major complications were defined as Clavien-Dindo grade IIIa or higher, because these complications require surgical, endoscopic, radiological, intensive care, or life-saving intervention.

### Outcome definitions

The primary outcome was delayed initiation of adjuvant therapy, defined as a time-to-treatment interval greater than 42 days from the date of total laryngectomy to the first day of postoperative radiotherapy or postoperative concurrent chemoradiotherapy. The 42-day threshold was selected because initiation of postoperative radiotherapy within 6 weeks after surgery is widely used as a clinically relevant benchmark in head and neck squamous cell carcinoma [12–15].

The time-to-treatment interval was calculated in calendar days as the difference between the date of total laryngectomy and the date of the first delivered fraction of postoperative radiotherapy. This variable was analyzed both as a continuous variable and as a categorical variable. The main categorical endpoint was delay greater than 42 days. A secondary categorical endpoint was delay greater than 56 days, selected to identify patients with more prolonged delay beyond the recommended postoperative treatment window.

Secondary outcomes included median and mean time-to-treatment interval according to complication status, proportion of patients with delay greater than 42 days and greater than 56 days according to complication status, time-to-treatment interval according to specific complication type, and the association between complication severity and treatment delay.

### Data management and quality control

Before analysis, the database was reviewed for completeness and internal consistency. Patient identifiers were checked to exclude duplicate records. Dates of surgery and adjuvant therapy initiation were verified, and the time-to-treatment interval was recalculated from source dates. Categorical variables were standardized before database locking, including complication labels, smoking status, and binary complication indicators. Records without a valid patient identifier or without sufficient information to calculate the primary outcome were excluded from the analytic dataset.

No statistical imputation was performed for missing data. Analyses were conducted using complete available data for each variable. Variables with substantial missingness were not used in multivariable models. When the reason for delay was not explicitly documented in the medical record, it was not inferred retrospectively.

For variables included in the primary analyses, date of surgery, date of adjuvant therapy initiation, time-to-treatment interval, postoperative complication status, age, sex, BMI, smoking status, T category, N category, and AJCC stage were available for all patients in the analytic cohort. Variables with incomplete availability, including detailed pathological risk factors, adjuvant treatment subtype, radiotherapy dose, number of fractions, nutritional biomarkers, and documented reason for delay, were not included in multivariable or inferential analyses.

### Statistical analysis

Continuous variables were summarized as mean and standard deviation or median and interquartile range, according to distribution. Normality was assessed using the Shapiro–Wilk test and visual inspection of histograms and Q-Q plots. Categorical variables were summarized as frequencies and percentages.

Baseline characteristics were compared between patients with and without postoperative complications. For continuous variables, the independent-samples *t*-test was used when the distribution was approximately normal, and the Mann–Whitney U test was used for non-normally distributed variables. For categorical variables, the chi-square test was used when expected cell counts were adequate, and the Fisher exact test was used when expected counts were small.

The association between postoperative complications and delayed adjuvant therapy greater than 42 days was evaluated using contingency table analysis. Because the endpoint was common and the association between complications and delay was very strong, odds ratios from standard logistic regression were considered potentially unstable and less clinically interpretable. Therefore, absolute risk differences and relative risks with 95% confidence intervals were reported as the primary effect measures for categorical delay outcomes. The Fisher exact test was used for between-group comparisons of delay thresholds.

Secondary analyses evaluated the association between postoperative complications and delay greater than 56 days. Because no patient without complications exceeded the 56-day threshold, relative risk and standard logistic regression estimates for this endpoint were considered non-estimable or unstable. Therefore, this endpoint was summarized using absolute risk difference and the Fisher exact test.

The time-to-treatment interval was compared as a continuous variable between patients with and without complications using the Mann–Whitney U test when non-normal distribution was present. Delay was also analyzed according to specific complication type, including pharyngocutaneous fistula, wound infection, dehiscence, hemorrhage, return to the operating room, and major complications. Since complication types were not mutually exclusive, complication-specific analyses were interpreted as exploratory.

The association between Clavien-Dindo grade and time-to-treatment interval was assessed using non-parametric correlation analysis and group comparison according to complication severity. Major complications were analyzed as a binary variable defined by Clavien-Dindo grade IIIa or higher. A two-sided *P* value less than 0.05 was considered statistically significant. Statistical analyses were performed using jamovi version 2.6.44 (The jamovi project, Sydney, Australia).

## RESULTS

### Cohort characteristics

A total of 203 patients undergoing primary total laryngectomy for laryngeal squamous cell carcinoma, subsequently initiating adjuvant therapy, and having a calculable time-to-treatment interval were included in the final analytic cohort. Postoperative complications before the initiation of adjuvant therapy were recorded in 49 patients (24.1%), whereas 154 patients (75.9%) had no documented postoperative complication.

The median age of the overall cohort was 65 years (IQR, 60–68), and most were male (175/203, 86.2%). Current smoking was documented in 166 patients (81.8%). Overall, 135 patients (66.5%) had T3 disease, and 68 patients (33.5%) had T4a disease.

Patients who developed postoperative complications had a slightly higher median BMI than those without complications (24.8 vs 24.2 kg/m^2^; *P* = 0.035). The proportion of T4a disease was numerically higher in the complication group (22/49, 44.9%) than in the no-complication group (46/154, 29.9%), although this difference did not reach conventional statistical significance (*P* = 0.058). No statistically significant differences were observed between groups for age, sex, smoking status, N3 disease, or AJCC stage distribution. Baseline characteristics are summarized in Table 1.

**Table 1:** Baseline characteristics according to postoperative complication status

Variable	Overall (*N* = 203)	No complications (*n* = 154)	Complications (*n* = 49)	*P* value
Age, years, median (IQR)	65 (60–68)	65 (60–68)	67 (62–69)	0.080
BMI, kg/m^2^, median (IQR)	24.4 (23.2–25.3)	24.2 (23.1–25.2)	24.8 (23.7–26.2)	0.035
Male sex	175 (86.2%)	130 (84.4%)	45 (91.8%)	0.239
Current smoker	166 (81.8%)	126 (81.8%)	40 (81.6%)	1.000
**T stage**				**0.058**
T3	135 (66.5%)	108 (70.1%)	27 (55.1%)	
T4a	68 (33.5%)	46 (29.9%)	22 (44.9%)	
N3 disease	22 (10.8%)	13 (8.4%)	9 (18.4%)	0.065
**AJCC stage**				**0.128**
Stage III	18 (8.9%)	15 (9.7%)	3 (6.1%)	
Stage IVA	163 (80.3%)	126 (81.8%)	37 (75.5%)	
Stage IVB	22 (10.8%)	13 (8.4%)	9 (18.4%)	

Data are *n* (%) unless otherwise indicated. *P* values were calculated using the Mann–Whitney U test for continuous variables and the chi-square or Fisher exact test for categorical variables. AJCC, American Joint Committee on Cancer; BMI, body mass index; IQR, interquartile range.

### Postoperative complications and severity profile

Among the 49 patients with postoperative morbidity, complication categories were recorded as patient-level binary variables and were not mutually exclusive. Therefore, a patient could contribute to more than one complication-type category. Pharyngocutaneous fistula was the most frequent complication, occurring in 39 patients (19.2% of the total cohort and 79.6% of patients with complications). Wound infection was recorded in 13 patients, wound/flap dehiscence in 11, and postoperative hemorrhage in 5.

Surgical reintervention or return to the operating room was required in 22 patients (10.8% of the full cohort and 44.9% of the complication group). According to the highest Clavien-Dindo grade, 19 patients had grade II complications, seven had grade IIIa complications, 19 had grade IIIb complications, three had grade IVa complications, and one had grade IVb complications. Major complications, defined as Clavien-Dindo grade IIIa or higher, occurred in 30 patients (14.8% of the total cohort and 61.2% of the complication group). No Clavien-Dindo grade V event was recorded. The complication profile is presented in Table 2.

**Table 2:** Postoperative complication profile

Variable	*n* (% of total cohort, *N* = 203)	% of complication group (*n* = 49)
Any postoperative complication	49 (24.1%)	100.0%
Pharyngocutaneous fistula	39 (19.2%)	79.6%
Wound infection	13 (6.4%)	26.5%
Wound/flap dehiscence	11 (5.4%)	22.4%
Postoperative hemorrhage	5 (2.5%)	10.2%
Return to operating room	22 (10.8%)	44.9%
**Clavien-Dindo grade**		
Grade II	19 (9.4%)	38.8%
Grade IIIa	7 (3.4%)	14.3%
Grade IIIb	19 (9.4%)	38.8%
Grade IVa	3 (1.5%)	6.1%
Grade IVb	1 (0.5%)	2.0%
Major complication (Clavien-Dindo ≥ IIIa)	30 (14.8%)	61.2%

Complication-type categories are not mutually exclusive; a patient with more than one complication may contribute to more than one complication-type row. Therefore, complication-type counts should not be summed to derive the number of patients with complications. Major complications were defined as Clavien-Dindo grade IIIa or higher, based on the highest grade recorded for each patient. CD, Clavien-Dindo.

### Impact of postoperative complications on time to adjuvant therapy

The overall median time-to-treatment interval from total laryngectomy to the start of adjuvant therapy was 42 days (IQR, 42–48), with a mean of 46.0 ± 7.8 days. Patients with postoperative complications had a markedly longer time-to-treatment interval than patients without complications. The median interval was 56 days (IQR, 52–62) in the complication group compared with 42 days (IQR, 42–42) in the no-complication group (*P* < 0.001). The corresponding mean values were 57.3 ± 8.5 days and 42.4 ± 1.9 days, respectively.

Delayed initiation of adjuvant therapy beyond 42 days occurred in 77/203 patients (37.9%). This endpoint was reached by 47/49 patients (95.9%) in the complication group and by 30/154 patients (19.5%) in the no-complication group (Fisher exact test, *P* < 0.001). In unadjusted contingency analysis, postoperative complications were associated with substantially higher odds of delay beyond 42 days (OR = 97.1; 95% CI, 22.3–422.5).

More prolonged delay beyond 56 days was observed exclusively in patients with postoperative complications. Overall, 23/203 patients (11.3%) exceeded the 56-day threshold. This included 23/49 patients (46.9%) in the complication group and 0/154 patients (0.0%) in the no-complication group (Fisher exact test, *P* < 0.001). Time-to-treatment outcomes according to complication status are summarized in Table 3 and illustrated in Figures 1 and 2.

**Table 3:** Time-to-adjuvant therapy according to postoperative complication status

Outcome	Overall	No complications	Complications	*P* value
Patients, *n*	203	154	49	
Time-to-treatment interval, days, mean ± SD	46.0 ± 7.8	42.4 ± 1.9	57.3 ± 8.5	<0.001
Time-to-treatment interval, days, median (IQR)	42 (42–48)	42 (42–42)	56 (52–62)	<0.001
Time-to-treatment interval, days, range	38–80	38–51	42–80	
Delay >42 days	77 (37.9%)	30 (19.5%)	47 (95.9%)	<0.001
Delay >56 days	23 (11.3%)	0 (0.0%)	23 (46.9%)	<0.001

The time-to-treatment interval was calculated in calendar days from total laryngectomy to the first day of postoperative radiotherapy or concurrent chemoradiotherapy. *P* values were calculated using the Mann–Whitney U test for continuous time-to-treatment comparisons and the Fisher exact test for delay thresholds. IQR, interquartile range; SD, standard deviation.

**Figure 1:**
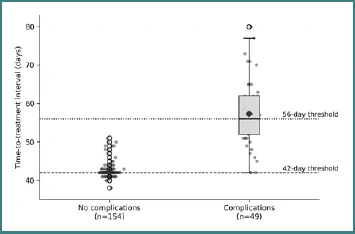
Distribution of the time-to-treatment interval according to postoperative complication status. Dashed and dotted horizontal lines indicate the 42-day and 56-day thresholds, respectively

**Figure 2:**
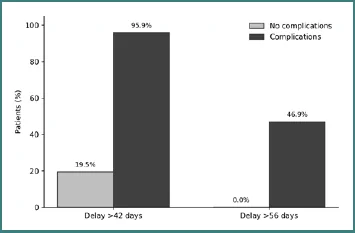
Proportion of patients exceeding 42-day and 56-day adjuvant therapy delay thresholds according to postoperative complication status

### Delay according to complication type and Clavien-Dindo severity

In complication-specific exploratory analyses, pharyngocutaneous fistula represented the most frequent complication associated with delayed adjuvant therapy. Among patients with pharyngocutaneous fistula, the median time-to-treatment interval was 58 days (IQR, 53–62); 38/39 patients (97.4%) exceeded 42 days and 20/39 (51.3%) exceeded 56 days.

The longest median time-to-treatment intervals were observed in patients with postoperative hemorrhage (65 days), wound/flap dehiscence (63 days), return to the operating room (61.5 days), and major complications (61 days). All patients with hemorrhage, wound/flap dehiscence, or return to the operating room exceeded the 42-day threshold. Delay beyond 56 days occurred in 5/5 patients with hemorrhage, 8/11 with wound/flap dehiscence, and 17/22 with return to the operating room.

Complication severity was also associated with the magnitude of delay. Patients with major complications had a longer median time-to-treatment interval than patients with minor Clavien-Dindo grade II complications (61 vs 53 days; *P* < 0.001). Delay beyond 56 days occurred in 20/30 patients (66.7%) with major complications compared with 3/19 patients (15.8%) with minor complications (Fisher exact test, *P* = 0.001). Across the complication group, higher Clavien-Dindo grade correlated with a longer time-to-treatment interval (Spearman ρ = 0.65; *P* < 0.001). Complication-specific delay patterns are summarized in Table 4 and Figure 3.

**Table 4:** Time-to-treatment interval and delay thresholds according to complication type and severity

Category	*n*	Median TTI, days (IQR)	Mean TTI, days ± SD	Delay >42 days	Delay >56 days
No complication	154	42 (42–42)	42.4 ± 1.9	30 (19.5%)	0 (0.0%)
Any complication	49	56 (52–62)	57.3 ± 8.5	47 (95.9%)	23 (46.9%)
Pharyngocutaneous fistula	39	58 (53–62)	58.5 ± 8.7	38 (97.4%)	20 (51.3%)
Wound infection	13	53 (51–58)	55.1 ± 7.1	12 (92.3%)	5 (38.5%)
Wound/flap dehiscence	11	63 (57–65)	62.1 ± 9.3	11 (100.0%)	8 (72.7%)
Postoperative hemorrhage	5	65 (61–73)	67.4 ± 7.3	5 (100.0%)	5 (100.0%)
Return to operating room	22	62 (59–69)	63.4 ± 7.7	22 (100.0%)	17 (77.3%)
Minor complication (Clavien-Dindo II)	19	53 (50–54)	52.1 ± 4.9	18 (94.7%)	3 (15.8%)
Major complication (Clavien-Dindo ≥ IIIa)	30	61 (55–65)	60.7 ± 8.6	29 (96.7%)	20 (66.7%)

Complication-specific categories are not mutually exclusive. Patients with multiple complications contribute to each applicable complication-type row. IQR, interquartile range; SD, standard deviation; TTI, time-to-treatment interval.

**Figure 3:**
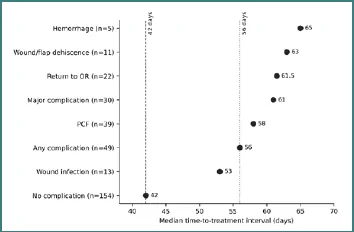
Median time-to-treatment interval according to complication type and severity. Vertical reference lines indicate the 42-day and 56-day thresholds. Complication-specific categories are not mutually exclusive. PCF, pharyngocutaneous fistula.

### Effect estimates for delayed adjuvant therapy

Because the primary endpoint was frequent and the association between postoperative complications and delay beyond 42 days was very strong, standard logistic regression produced unstable odds ratio estimates with wide confidence intervals. Therefore, risk differences and relative risks were considered more clinically interpretable effect measures.

For delay beyond 42 days, postoperative complications were associated with an absolute risk difference of 76.4 percentage points (95% CI, 68.1–84.8) and a relative risk of 4.92 (95% CI, 3.55–6.82). For delay beyond 56 days, the absolute risk difference was 46.9 percentage points (95% CI, 33.0–60.9). Relative risk was not estimated for the 56-day threshold because no patient without complications exceeded this cutoff, resulting in a zero-event cell. Fisher exact testing remained statistically significant for both delay thresholds (*P* < 0.001; Table 5).

**Table 5:** Effect estimates for postoperative complications and delayed adjuvant therapy

Endpoint	No complications (*n* = 154)	Complications (*n* = 49)	Absolute risk difference	Relative risk	*P* value
Delay >42 days	30/154 (19.5%)	47/49 (95.9%)	76.4 (95% CI, 68.1–84.8)	4.92 (95% CI, 3.55–6.82)	<0.001
Delay >56 days	0/154 (0.0%)	23/49 (46.9%)	46.9 (95% CI, 33.0–60.9)	Not estimable (zero events)	<0.001

*P* values were calculated using the Fisher exact test. CI, confidence interval; RR, relative risk. Relative risk was not estimated for delay >56 days because no patient without complications exceeded this threshold.

## DISCUSSION

The present study evaluated whether postoperative complications after primary total laryngectomy were associated with delayed initiation of adjuvant therapy. The main finding was that patients who developed postoperative complications had a substantially longer time-to-treatment interval than those with an uncomplicated postoperative course. Median time to adjuvant therapy was 56 days in the complication group compared with 42 days in the no-complication group, and delay beyond 42 days occurred in 95.9% of patients with complications compared with 19.5% of patients without complications. More prolonged delay beyond 56 days was observed only in patients with postoperative complications. These findings suggest that postoperative morbidity after total laryngectomy is not only a surgical outcome but also a clinically relevant factor that may interfere with the planned oncological treatment sequence.

The clinical relevance of this association is based on the importance of the 6-week postoperative window for radiotherapy initiation in head and neck squamous cell carcinoma. Initiation of postoperative radiotherapy within 42 days after surgery is widely used as a quality-of-care benchmark in head and neck cancer, and several studies have associated longer surgery-to-radiotherapy intervals with worse oncological outcomes [13,15]. In a National Cancer Database analysis including patients with head and neck squamous cell carcinoma treated with surgery followed by adjuvant radiation, Harris *et al*. reported that a shorter interval from surgery to radiation therapy was associated with improved survival, with 42 days used as a clinically relevant reference threshold [21]. Similarly, the systematic review by Graboyes *et al*. found that treatment delays across the head and neck cancer care continuum, including the interval between surgery and postoperative radiotherapy, were associated with poorer survival in several included studies [22]. The present study did not evaluate recurrence, locoregional control, disease-free survival, or overall survival; therefore, it cannot conclude that complications directly worsened oncological outcomes. However, the high proportion of patients with complications exceeding the 42-day threshold is clinically meaningful because it identifies a plausible mechanism through which postoperative morbidity may compromise the delivery of time-sensitive adjuvant treatment.

Our results are consistent with previous research showing that delays in postoperative radiotherapy are multifactorial. Systematic reviews have identified patient-level, disease-level, treatment-level, and health-system factors associated with delayed postoperative radiotherapy in head and neck cancer [14,18]. In the qualitative study by Graboyes *et al.*, postsurgical sequelae that interrupt the tight treatment timeline were identified as one of the relevant barriers to timely postoperative radiotherapy, alongside insufficient coordination during care transitions, fragmentation of care, travel burden, and inadequate education about the importance of timely adjuvant therapy [23]. The present study adds to this literature by focusing specifically on a homogeneous cohort of patients undergoing primary total laryngectomy and by quantifying the magnitude of delay associated with postoperative complications in this surgical population.

Total laryngectomy is known to be associated with substantial postoperative morbidity. Goepfert *et al*. reported that complications after total laryngectomy are associated with increased hospital length of stay and readmission, confirming that these patients represent a high-risk surgical population [4]. In our cohort, the overall complication rate was 24.1%, which is within the broad range reported in the literature for laryngectomy-associated morbidity, although direct comparison between studies is limited by differences in patient selection, surgical technique, salvage versus primary setting, reconstruction type, complication definitions, and follow-up duration [4–9]. Importantly, our study included only primary total laryngectomy patients with an indication for adjuvant therapy and excluded salvage laryngectomy cases, which improves internal homogeneity but limits comparability with studies including previously irradiated patients.

Pharyngocutaneous fistula was the most frequent complication associated with delayed adjuvant therapy in our cohort. This finding is biologically and clinically plausible. PCF represents a failure of pharyngeal closure, exposes cervical tissues to salivary contamination, frequently requires prolonged enteral feeding and repeated wound care, and may be complicated by infection, dehiscence, bleeding, or need for surgical repair [5–9]. Previous studies have consistently identified PCF as one of the most important complications after total laryngectomy, although reported incidence and risk factors vary across cohorts [5–9]. A recent narrative review emphasized that PCF remains one of the most frequent and serious complications after total laryngectomy and may delay the initiation of adjuvant treatment when postoperative radiotherapy is indicated [24]. The present data support this clinical assumption by showing that patients with PCF frequently exceeded the 42-day adjuvant treatment window.

Wound infection and wound or flap dehiscence also deserve attention because they may be part of the same postoperative wound-healing continuum as PCF. In clinical practice, these complications often coexist rather than occur as isolated events. Infection may weaken wound integrity, dehiscence may expose deeper tissues and facilitate salivary contamination, and PCF may perpetuate local inflammation and bacterial colonization. This overlap is important when interpreting complication-specific analyses. The present study classified complications individually, but categories were not mutually exclusive. Therefore, the delay associated with a specific complication type should be interpreted as an exploratory signal rather than as an independent causal estimate.

Postoperative hemorrhage, return to the operating room, and major complications defined by Clavien-Dindo grade IIIa or higher were less frequent than PCF but were associated with particularly prolonged time-to-treatment intervals. This is clinically expected because severe complications often require additional procedures, repeated anesthesia, intensive postoperative surveillance, transfusion, vascular control, flap revision, or prolonged hospitalization. In such cases, the recovery clock may effectively restart after reintervention, making timely radiotherapy more difficult to achieve. However, because the number of patients with hemorrhage and some other severe complication types was relatively small, these findings should be considered hypothesis-generating. Larger multicenter studies are needed to determine whether specific severe complications independently predict prolonged adjuvant treatment delay after adjustment for tumor stage, reconstruction type, comorbidities, and institutional factors.

An important observation is that 19.5% of patients without postoperative complications still initiated adjuvant therapy after 42 days. This finding indicates that postoperative complications are not the only source of delay. Even in uncomplicated cases, delays may result from referral timing, pathology reporting, tumor board scheduling, radiation oncology consultation, dental evaluation, CT simulation, treatment planning, patient adherence, transport difficulties, or resource availability. This is consistent with previous work showing that timely postoperative radiotherapy depends on coordinated transitions between surgery and radiation oncology rather than on surgical recovery alone [14,18,23]. Therefore, while preventing complications is important, it should be integrated into a broader institutional strategy aimed at reducing all avoidable delays between surgery and adjuvant treatment.

The clinical implication of the present study is that preventing and rapidly managing postoperative complications after total laryngectomy should be considered part of oncological quality improvement. This does not mean that adjuvant radiotherapy should be started in the presence of an unsafe wound, active salivary leakage, uncontrolled infection, or unstable postoperative condition. Rather, it means that surgical teams should anticipate which patients are at high risk for complications, optimize modifiable risk factors before surgery, monitor wound healing aggressively after surgery, and coordinate early with radiation oncology even before complete wound healing when appropriate. Such coordination may reduce non-medical waiting time once the patient becomes fit for radiotherapy.

Several practical strategies may emerge from these findings. Preoperative evaluation should include nutritional assessment, correction of anemia when feasible, diabetes optimization, cardiovascular risk assessment, smoking cessation counseling, and careful surgical planning. Intraoperatively, closure technique and reconstructive strategy should be individualized according to patient risk factors, tissue quality, and defect extent. Postoperatively, early recognition of salivary leakage, infection, or dehiscence may allow faster escalation from conservative management to surgical repair when conservative measures are unlikely to succeed. At the care-pathway level, early radiation oncology referral, prospective scheduling of postoperative review, standardized communication between surgical and radiation teams, and patient navigation may help preserve the adjuvant treatment window [23,25].

The present study has several strengths. First, it focused on a specific and clinically relevant population: patients undergoing primary total laryngectomy for laryngeal squamous cell carcinoma with an indication for adjuvant therapy. This avoids some of the heterogeneity present in broader head and neck cancer studies that combine different tumor sites and surgical procedures. Second, the primary outcome was based on objective calendar dates: date of surgery and date of first adjuvant radiotherapy fraction. Third, the study used a clinically meaningful 42-day threshold and also evaluated a more prolonged delay threshold of 56 days. Fourth, the analysis included both overall postoperative complication status and exploratory complication-specific patterns, allowing the results to be interpreted from both statistical and clinical perspectives. Finally, the study provides data from an Eastern European tertiary center, a setting that is underrepresented in the literature on adjuvant treatment timeliness after head and neck cancer surgery.

The limitations of this study should be acknowledged. Its retrospective design limits the ability to establish causality. Although complications occurred before the initiation of adjuvant therapy, residual confounding cannot be excluded. Patients with complications may have differed from those without complications in ways not fully captured in the database, including frailty, socioeconomic status, access to radiotherapy services, wound care logistics, family support, or unmeasured comorbidities. The single-center design may also limit generalizability, because treatment pathways, radiotherapy access, referral patterns, and postoperative care protocols vary between institutions.

Another limitation is that the study did not evaluate long-term oncological outcomes. Therefore, although delayed adjuvant therapy is clinically relevant and supported by previous literature, this cohort cannot determine whether the observed delays translated into higher rates of recurrence, lower locoregional control, or worse survival. Future studies should link postoperative complications, time-to-adjuvant therapy, treatment package time, recurrence, disease-free survival, overall survival, and quality-of-life outcomes. Such data would clarify whether preventing complications after total laryngectomy improves oncological outcomes through preservation of the postoperative adjuvant therapy window.

Complication-specific analyses should also be interpreted cautiously. Several complications overlapped in the same patients, and some events, such as postoperative hemorrhage, occurred in relatively small numbers. Therefore, the study is not powered to determine whether each individual complication independently predicts delay. Similarly, Clavien-Dindo grades were assigned retrospectively from available clinical records, which may introduce classification bias if documentation was incomplete. The analysis of major complications remains clinically useful, but prospective grading would provide more reliable severity assessment.

Finally, the study included only patients for whom the time-to-treatment interval could be calculated. If patients with severe postoperative complications never initiated adjuvant therapy, their exclusion may underestimate the true impact of postoperative morbidity on the adjuvant treatment pathway. This is an important consideration for future prospective studies, which should record not only delayed adjuvant therapy but also omission of planned adjuvant therapy and the documented reasons for omission.

Overall, the present findings support the concept that postoperative complications after total laryngectomy are closely linked to delayed initiation of adjuvant therapy. The data do not prove that complications directly worsen survival, but they show that complications frequently push patients beyond a widely used postoperative treatment window. This reinforces the need to view complication prevention, early wound management, and multidisciplinary coordination as components of oncological care rather than as isolated surgical concerns.

## CONCLUSION

Postoperative complications following primary total laryngectomy were strongly associated with delayed initiation of adjuvant therapy in this cohort of patients with laryngeal squamous cell carcinoma. Patients who developed complications experienced substantially longer time-to-treatment intervals and were significantly more likely to exceed the widely accepted 42-day postoperative treatment window. Pharyngocutaneous fistula, wound-related complications, and major postoperative adverse events were particularly associated with prolonged delays, highlighting the importance of adequate wound healing in maintaining the planned oncological treatment pathway. Although this study did not evaluate long-term oncological outcomes, the observed delays are clinically relevant given the established association between postponed postoperative radiotherapy and poorer outcomes reported in the head and neck cancer literature. These findings support the concept that prevention and early management of postoperative complications should be regarded not only as surgical objectives but also as components of high-quality oncological care. Multidisciplinary coordination between surgeons, radiation oncologists, and supportive care teams may help preserve the recommended postoperative treatment timeline and reduce avoidable delays. Future prospective studies should investigate whether complication-related delays translate into differences in recurrence rates, disease-free survival, overall survival, and patient-reported outcomes.

## Data Availability

The data analyzed during the current study were obtained retrospectively from hospital medical records and maintained in an anonymized research database. The datasets are not publicly available to protect patient confidentiality and comply with applicable data protection regulations.
